# Annotations

**Published:** 1908-07-04

**Authors:** 


### Annotations.

### The Age of Forty.

When Professor Osler some few years ago in the course of a speech ventured the opinion that "the effective moving vitalising work of the world is done between the ages of twenty-five and forty," his words were instantly taken up and discussed by the Press throughout England and America, and even on the Continent; and the echoes of the discussion which he provoked have hardly yet died away. Indeed, to the lay public the name of Osler is best known in connection with the "too old at forty idea." No one, we believe, regrets this association more than Professor Osler. The words we quote were spoken among many which were clearly not meant to be taken seriously; and, although there is some measure of truth in them, we never believed that their author intended them as anything more than *obiter dicta*. The latest pronouncement upon the subject is made by Dr. Newman Dorland in the *Century*. Here will be found an elaborate and exhaustive account of what the world owes to men above the age of forty. Dr. Dorland is a capable apologist, and his vindication of middle age carries with it conviction. He has ransacked every field of intellectual activity, and his array of discoveries, books, works of art, and notable advances of every kind, which have been made throughout the centuries by great men after they had passed the age of forty, is a monument of industry. It is really an astonishing tribute to Professor Osler that a few chance words of his spoken several years ago should still be capable of goading clever men into elaborate historical and statistical research. According to Dr. Dorland—and life is too short to dispute the matter with him—the average age at which great men produce their masterpieces is for "workers" forty-seven, and for "thinkers" fifty-two. "Provided health and optimism remain," he says, "the man of fifty can command success as readily as a man of thirty." That is indeed comforting; but the proviso is a big one.

### The Birthday Honours.

Several members of the medical profession have received well-deserved honours on the occasion of his Majesty's official birthday. Two baronetcies have been conferred upon distinguished representatives of medicine and surgery respectively, and all will agree in congratulating Sir Lauder Brunton and Professor Watson Cheyne upon their new distinctions. Sir Lauder Brunton is eminent alike as a physician and as a pharmacologist; while the selection of Mr. Watson Cheyne for titular distinction is justified by his surgical attainments and by his services to the country during the South African war. St. Bartholomew's Hospital and King's College Hospital each gain reflected honour from these awards, while Edinburgh University can claim both the recipients as former students. Mr. William Henry Power, whose excellent work as medical officer to the Local Government Board is well known, has been promoted to the rank of K.C.B., and knighthoods have been conferred upon Colonel David Bruce and Dr. Robert William Burnet. Dr. Burnet is honorary physician to the Prince of Wales, and was in close attendance upon his friend, the late Premier, during his last illness; while Colonel David Bruce's invaluable contributions to medical knowledge and treatment, more especially in connection with Malta fever, fully entitle him to his new distinction, which will be applauded throughout the Royal Army Medical Corps and the profession generally. Two further Birthday honours for medical men are to be recorded: Lieutenant-Colonel Johnston Shearer has received the Companionship of the Bath and Lieutenant-Colonel Francis Frederic Perry becomes a Companion of the Order of the Indian Empire. Both these officers belong to the Indian medical service, and Colonel Perry is honorary surgeon to the Viceroy and Principal of the Medical College, Lahore. The distribution of medical Birthday honours shows a nice discrimination into the many aspects of professional work and merit. Thus the pioneer work of medical research, no less than clinical eminence and distinguished official service, receives public recognition from the Sovereign.

### Art and Prudery.

A certain London evening paper has lately broken out into a ludicrous excess of moral censorship. It seems as though a member of the staff of this periodical, whilst walking down the Strand a week or two ago, happened to look up at the new buildings of the British Medical Association, whose frontage had just been disclosed to the public view by the removal of part of the hoarding. The distant vision of some of the eighteen statues which adorn these premises at once inspired him to write an indignant protest against their hideous immorality. Although the silly season is not yet due, the editor seems to have discerned by a flash of journalistic genius that the pose of outraged modesty has distinct possibilities even in the middle of June. From this point of view the editor and his staff must be congratulated on their foresight and enterprise, for they have provoked the man in the street to look for offence where none is, and have set the town talking about the "Strand statues." And what, after all, are these statues? They are single, symbolical figures, some draped, some nude, placed at a height of forty or fifty feet above the ground, and totally devoid, in our opinion, of indecency, or immorality. Personally, we do not admire them as works of art, and, in spite of the warm approval of many distinguished artists and critics, we maintain that several of them are ugly. But to find the remotest suggestion of impurity in these figures seems to us to presuppose in the mind of the observer, if not impurity, at least a talent for smelling out unintentional nastiness, which is even worse than prudery. A special report on these statues in the *Times*, together with a sharp rebuke in a leading article, only stirred up further insinuations; but we are glad to learn that the Council of the British Medical Association decided on June 24 to allow the work to continue, and we hope that nothing will induce it to take any further notice of what seems to be little more than a newspaper campaign.

